# Short-Term Exposure to Ambient Air Pollution and Increased Emergency Room Visits for Skin Diseases in Beijing, China

**DOI:** 10.3390/toxics9050108

**Published:** 2021-05-12

**Authors:** Wanzhou Wang, Wenlou Zhang, Jingjing Zhao, Hongyu Li, Jun Wu, Furong Deng, Qingbian Ma, Xinbiao Guo

**Affiliations:** 1 Department of Occupational and Environmental Health Sciences, School of Public Health, Peking University, Beijing 100191, China; 1610306235@pku.edu.cn (W.W.); 1410306229@pku.edu.cn (W.Z.); hylee1030@126.com (H.L.); guoxb@bjmu.edu.cn (X.G.); 2 Emergency Department, Peking University Third Hospital, Beijing 100191, China; jeanzhao123@163.com (J.Z.); woshiwujun0613@sina.com (J.W.)

**Keywords:** air pollution, particulate matter, emergency room visits, skin diseases, dermatitis, eczema, urticaria

## Abstract

Skin diseases have become a global concern. This study aims to evaluate the associations between ambient air pollution and emergency room visits for skin diseases under the background of improving air quality in China. Based on 45,094 cases from a general hospital and fixed-site monitoring environmental data from 2014–2019 in Beijing, China, this study used generalized additive models with quasi-Poisson regression to estimate the exposure–health associations at lag 0–1 to lag 0–7. PM_2.5_ and NO_2_ exposure were associated with increased emergency room visits for total skin diseases (ICD10: L00-L99). Positive associations of PM_2.5_, PM_10_, O_3_ and NO_2_ with dermatitis/eczema (ICD-10: L20–30), as well as SO_2_ and NO_2_ with urticaria (ICD-10: L50) visits were also found. For instance, a 10 μg/m^3^ increase in PM_2.5_ was associated with increases of 0.7% (95%CI: 0.2%, 1.2%) in total skin diseases visits at lag 0–5 and 1.1% (95%CI: 0.6%, 1.7%) in dermatitis/eczema visits at lag 0–1, respectively. For PM_2.5_, PM_10_ and CO, stronger annual associations were typically observed in the high-pollution (2014) and low-pollution (2018/2019) years. For instance, a 10 μg/m^3^ increase in PM_2.5_ at lag 0–5 was associated with increases of 1.8% (95%CI: 1.0%, 2.6%) and 2.3% (95%CI: 0.4%, 4.3%) in total skin disease visits in 2014 and 2018, respectively. Our study emphasizes the necessity of controlling the potential health hazard of air pollutants on skin, although significant achievements in air quality control have been made in China.

## 1. Introduction

Ambient air pollution is associated with exacerbated disease burden in the global scale [1]. Short- and long-term exposures to ambient air pollution are associated with increased all-cause mortality and risks of cardiovascular diseases and asthma at the population level [2,3,4]. Skin diseases have become a major challenge for global public health due to their high prevalence and severe disease burden. Among the top 50 most prevalent diseases, eight were skin and subcutaneous diseases [5], affecting one in four individuals worldwide in 2019 [6], with a rapid increase of over 24.0% compared to 1990 [7]. In 2019, skin and subcutaneous diseases accounted for 42.9 million disability-adjusted life years (DALYs) globally, among which dermatitis and eczema contributed to the greatest proportion [6]. In China, there were more than 369 million skin disease cases, contributing to 8.3 million DALYs in 2019 [6]. The concurrent increasing trend of skin disease prevalence and ambient air pollution brings novel insights into its environmental etiology [8,9]. Several epidemiological studies have also linked exposure to air pollution, especially particulate matter (PM) to increased risks of dermatitis, eczema [10,11,12,13,14], acne vulgaris [15], etc., but yielded inconclusive findings partly attributable to the variations across populations and regions with different environment backgrounds [16].

In 2013, Air Pollution Prevention and Control Action Plan (APPCAP) was launched to overcome the air pollution challenge in China with a focus on several key regions [17], among which the Beijing–Tianjin–Hebei region was of great concern due to its severe pollution [18]. As a key prevention and control area, Beijing additionally initiated the Clean Air Action Plan from 2013–2017, causing remarkable reductions in air pollutants, except for ozone (O_3_), since 2013 [19]. This provides a good opportunity to evaluate the potential health effects of air pollutants at different concentrations [20].

There were two major limitations of recent studies. First, most previous evidence was based on outpatient visits, which were relatively less sensitive to capture the acute effects of air pollutants. Meanwhile, to date, the potential effect of the constant improvement of air quality on skin diseases remains unexplored. Therefore, this study investigated the association between short-term exposure to ambient air pollution and emergency room visits for skin diseases, a sensitive measure for the acute onset of skin diseases at the population level. We also, for the first time, explored its temporal variability from 2014–2019, a time period showing remarkable and continuous improvement in air quality in Beijing, China.

## 2. Materials and methods

### 2.1. Study Design and Population

The present time-series study was conducted based on a population derived from the emergency room visit data of Peking University Third Hospital (https://www.puh3.net.cn/englishweb/index.shtml, accessed on 1 August 2020), which is one of the largest tertiary general hospitals in Beijing. In 2019, the hospital provided more than 300,000 emergency medical services for people in all age groups and with different kinds of demographical characteristics. The patients were similar to those of other public hospitals, and accounted for about 15~20% of the total emergency cases in Beijing.

We extracted daily hospital emergency room visits for skin diseases from January 2014 through December 2019 recorded in the hospital information system (HIS) coded as the International Classification of Disease, 10th revision (ICD-10): L00-L99. The emergency room visits for two major skin diseases, dermatitis/eczema (ICD-10: L20–30) and urticaria (ICD-10: L50) were also extracted [21].

The data used for this study were collected for administrative purposes without individual identifiers, and thus could be exempt from the Institutional Review Board.

### 2.2. Air Pollutants and Meteorological Data

We included six major ambient air pollutants, including the fine particles (PM_2.5_), inhalable particles (PM_10_), nitrogen dioxide (NO_2_), sulfur dioxide (SO_2_), O_3_ and carbon monoxide (CO). The Chinese Air Quality Online Monitoring and Analysis Platform (https://www.aqistudy.cn/, accessed on 5 August 2020) provided hourly concentrations of the six air pollutants in 35 fixed-site stations in Beijing. Daily average concentrations of five ambient pollutants (PM_2.5_, PM_10_, NO_2_, SO_2_ and CO) and 8 h maximum concentration of O_3_ during the study period were calculated, as a proxy for population exposure, in line with previous time-series studies [10,11]. The National Meteorological Information Center (http://data.cma.cn/, accessed on 5 August 2020) provided daily meteorological data such as temperature and relative humidity (RH) of 20 national observatories in Beijing. The numbers (proportion) of missing data were 7 (0.32%), 42 (1.92%), 18 (0.82%), 1 (0.05%), 1 (0.05%) and 1 (0.05%) for PM_2.5_, PM_10_, O_3_, SO_2_, NO_2_ and CO, respectively, and they were excluded from the analysis.

### 2.3. Statistical Analysis

To capture the short-term cumulative lagged associations between ambient air pollution and emergency room visits for skin diseases, 2- to 8-day moving average concentrations (lag 0–1 to lag 0–7, the average concentration of the present day and previous 1–7 days) of the six pollutants were generated and assigned to the daily visit data. The exposure metrics were selected based on previous time-series studies [22,23]. First, we conducted a time-series analysis to estimate the overall associations over the whole study period (2014–2019). Generalized additive models with quasi-Poisson regression [24] were performed using the following formula:

Log[*E*(*Y_t_*)] = α + βZ*_t_* + DOW + Holiday + ns (calendar time, df = 7 per year) + cb (T_t_, …, T_t−14_, df = 4) + ns (RH, df = 3)

where *t* represents the day of the observation; *E*(*Y_t_*) is the expected number of emergency room visits for skin diseases on day *t*; β is the regression coefficient of air pollutants; Z*_t_* is the moving average concentrations of ambient air pollutants at lag 0–1 to lag 0–7 for day *t*. DOW and Holiday are the category day of week and holiday variable included to account for temporal variations across weeks and holidays. The ns (calendar time, df = 7 per year) indicates a natural cubic spline function with 7 degrees of freedom per year for calendar time [3]. Temperature and RH were also used to adjust for potential non-linear and delayed confounding effects of weather conditions. Specifically, cb (T_t_, …, T_t−14_, df = 4) is a cross-basis function using the distributed lag nonlinear model (DLNM) with a maximum period of 14 days and 4 degrees of freedom for temperature [25], and ns (RH, df = 3) is a natural cubic spline function with 3 degrees of freedom for RH, consistent with previous studies [26]. Subgroup analyses stratified by individual age (≤18, 18–39 and ≥40 years), sex and season (warm season: May–Oct; cold season: Nov–Apr) were performed [27]. The inter-group differences were examined using the Z-test [3].

To further explore the temporal variability of the associations between ambient air pollutants and emergency room visits for skin diseases over the study period, we performed time-stratified analyses using interaction models, which included the main effect terms of pollutants and year, their multiplicative interaction terms, as well as aforementioned covariates. The yearly effect estimates were obtained from the regression coefficients of pollutants and interaction terms [26]. The interaction terms of pollutants and year were also examined to evaluate the differences by year [28,29].

Several additional analyses were used to evaluate the robustness of the results. First, two-pollutant models were conducted for each air pollutant by adjusting one other air pollutant with the same exposure metrics. Second, we used a different lag time of 30 days for temperature to account for its potential non-linear effects at a longer exposure metric, and different df values of 4–10 per year for calendar time, as used previously [26,30]. Third, we also conducted time-stratified analyses based on three time periods: 2014–2015, 2016–2017 and 2018–2019, considering that the annual estimates might be unstable, as reported previously [28]. In addition, we plotted the exposure–response curves to characterize the associations between air pollutants and daily emergency room visits for skin diseases at different exposure concentrations, where we replaced the linear term of air pollutants in the main model with a natural smoothing function with 3 dfs [31]. We also compared the main models and the non-linear models according to the minimized AIC criteria.

Results were expressed as the percentage changes and 95% confidence intervals (CIs) in the emergency room visits for skin diseases with a 10 μg/m^3^ increase in PM_2.5_, PM_10_, NO_2_, SO_2_, 8 h maximum O_3_ concentrations and an increase of 1 mg/m^3^ in CO concentration. All statistical analyses were performed using R software (Version 4.0.0) with “mgcv” and “dlnm” packages. A two-sided *p* < 0.05 was considered statistically significant.

## 3. Results

### 3.1. Descriptive Results

A total of 45,094 emergency room visits for skin diseases were identified in this study from 2014–2019, among which 16,891 (37.5%) were dermatitis/eczema cases, and 23,832 (52.8%) were urticaria cases (Table 1). Figure S1 presents the daily distribution of emergency room visits for total skin diseases, dermatitis/eczema and urticaria from 2014–2019.

**Figure 1:**
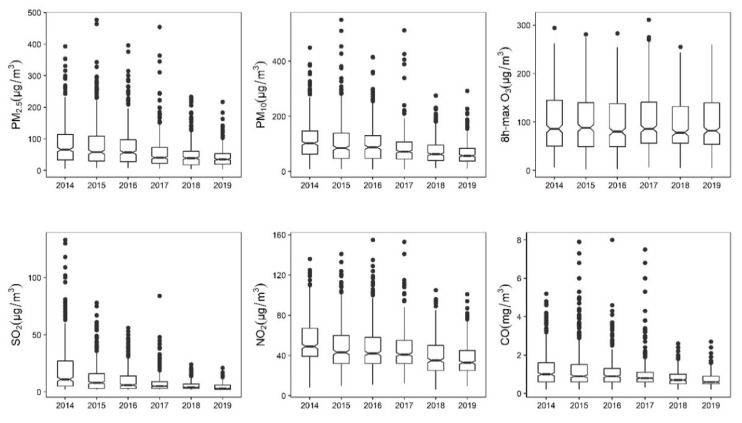
Annual distributions of daily average concentrations of ambient PM_2.5_, PM_10_, 8 h maximum O_3_, SO_2_, NO_2_ and CO in Beijing during the study period (2014–2019).

As shown in Figure 1, remarkable decreases were observed in PM_2.5_, PM_10_, NO_2_, SO_2_ and CO, but not 8 h maximum O_3_ from 2014–2019. Distributions of daily concentrations of the ambient air pollutants and meteorological variables during the study period are shown in Table S1. The results of Spearman correlation analysis between air pollutants and meteorological variables are presented in Table S2.

### 3.2. Associations between Ambient Air Pollutants and Emergency Room Visits for Skin Diseases

**Figure 2:**
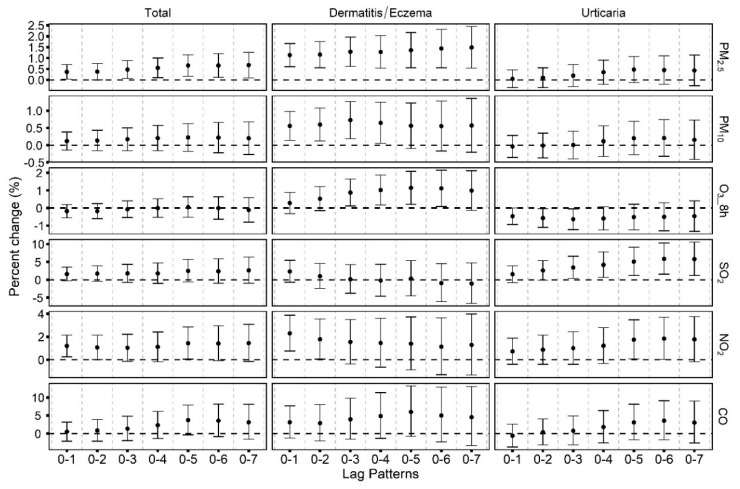
Overall percent changes and 95% confidence intervals (CIs) in daily emergency room visits for skin diseases per 10 μg/m^3^ increases in PM_2.5_, PM_10_, 8 h maximum O_3_, SO_2_, NO_2_ and 1 mg/m^3^ increase in CO with different time metrics (2014–2019).

As shown in Figure 2, short-term PM_2.5_ and NO_2_ exposures were associated with increased emergency room visits for total skin diseases. Positive associations of PM_2.5_, PM_10_, O_3_ and NO_2_ with dermatitis/eczema, as well as SO_2_ and NO_2_ with urticaria visits were also found. For instance, a 10 μg/m^3^ increase in PM_2.5_ was associated with increases of 0.7% (95%CI: 0.2%, 1.2%) in total skin disease visits at lag 0–5 and 1.1% (95%CI: 0.6%, 1.7%) in dermatitis/eczema visits at lag 0–1. A 10 μg/m^3^ increase in O_3_ at lag 0–5 was associated with a significant increase of 1.1% (95%CI: 0.2%, 2.1%) in dermatitis/eczema visits. In addition, a 10 μg/m^3^ increase in NO_2_ was associated with a significant increase of 1.2% (95%CI: 0.3%, 2.2%) in total skin disease visits.

**Figure 3:**
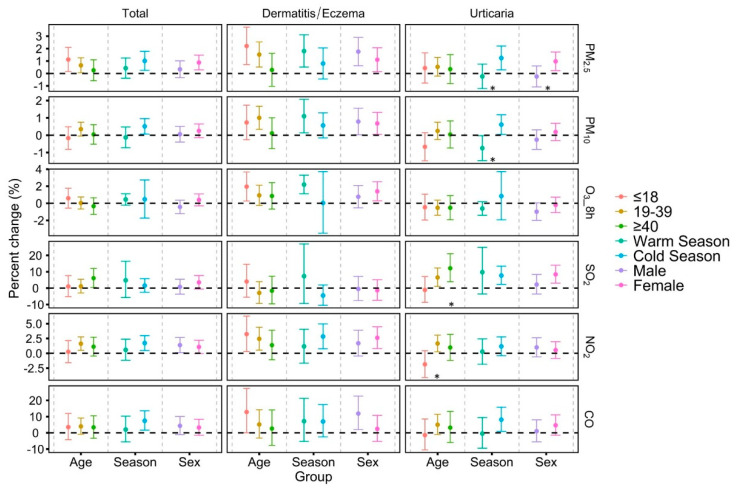
Percent changes and 95% confidence intervals (CIs) in daily emergency room visits for skin diseases per 10 μg/m^3^ increases in PM_2.5_ (lag 0–5), PM_10_ (lag 0–3), 8 h maximum O_3_ (lag 0–5), SO_2_ (lag 0–6), NO_2_ (lag 0–1) and 1 mg/m^3^ increase in CO (lag 0–5) from 2014–2019, stratified by age, season and sex. Note: Warm season: May–Oct; cold season: November–April. * *p* for subgroup differences < 0.05.

Figure 3 demonstrates the results of subgroup analyses stratified by age, sex and season, based on the time metric with the most significant effect estimate (with the smallest *p*-value) for each air pollutant. Generally, we did not find consistent differences in the effect estimates between different age, season and sex groups. The associations of SO_2_ and NO_2_ with urticaria were more significant in people aged ≥40 years compared to ≤18 years. Stronger associations were found between urticaria visits and PM_2.5_ and PM_10_ in the cold season. Meanwhile, female individuals were more susceptible to PM_2.5_ exposure for the urticaria visits. The results of other lag patterns (lag0–1~lag0–7) also highlighted these findings (Figures S2–S4).

### 3.3. Temporal Variability of the Associations between Air Pollutants and Emergency Room Visits for Skin Diseases

**Figure 4:**
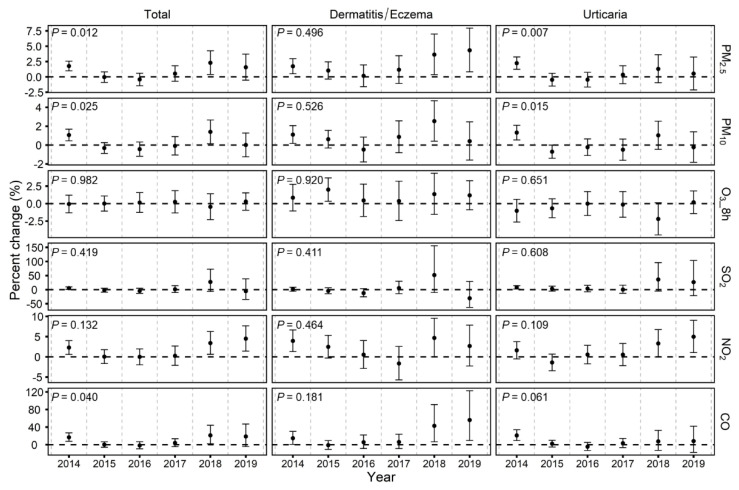
Annual percent changes and 95% confidence intervals (CIs) in daily emergency room visits for skin diseases along with a 10 μg/m^3^ increase in in PM_2.5_ (lag 0–5), PM_10_ (lag 0–3), 8 h maximum O_3_ (lag 0–5), SO_2_ (lag 0–6), NO_2_ (lag 0–1) and a 1 mg/m^3^ increase in CO (lag 0–5) from 2014–2019. Note: *p*-value indicates the results of the heterogeneity test on annual estimates based on multiplicative interaction terms of pollutants and year.

As displayed in Figure 4, stronger annual associations of PM_2.5_, PM_10_ and CO with emergency room visits for total skin diseases and urticaria were typically found in the high-pollution (2014) and low-pollution (2018/2019) years, while trends for dermatitis/eczema were less consistent. There were statistically significant differences by year for associations of PM_2.5_ and PM_10_ with emergency room visits for total skin diseases and urticaria, as well as CO with total skin diseases (*p* for interaction term of pollutant and year <0.05). For instance, along with a 10 μg/m^3^ increase in PM_2.5_ concentration at lag 0–5, we observed a significant increase of 1.8% (95%CI: 1.0%, 2.6%) in 2014, a weaker insignificant change of 0.5% (95%CI: −0.7%, 1.8%) in 2017 and a relapsed increase of 2.3% (95%CI: 0.4%, 4.3%) in 2018 for emergency room visits for total skin diseases in the interaction models (*p* for interaction term of pollutant and year = 0.012). Meanwhile, we did not observe apparent changes in the associations between skin disease visits and O_3_ and SO_2_ over the study period.

### 3.4. Additional Analyses

After additionally adjusting for co-pollutants and maximum lags for temperature (30 days), the associations were generally robust, although several estimates were attenuated (Tables S3 and S4). Analyses stratified by two-year periods showed a similar temporal variability feature (Figure S5). In addition, the results of exposure–response curves were generally consistent with the aforementioned findings over the whole study period (Figure S6). The comparison between main models and non-linear models showed that the main models performed better according to the minimized AIC criteria.

## 4. Discussion

Clarifying the potential environmental risk factors which mediate the acute onset of skin diseases is important for patient-centered prevention and control with public health relevance. To this end, this study evaluated the associations between short-term exposure to ambient air pollutants and emergency room visits for total skin diseases, dermatitis/eczema and urticaria in Beijing, China from 2014–2019. We found that short-term PM_2.5_ and NO_2_ exposures were associated with increased emergency room visits for total skin diseases. Meanwhile, positive associations of PM_2.5_, PM_10_, O_3_ and NO_2_ with dermatitis/eczema, as well as SO_2_ and NO_2_ with urticaria visits were also found. In addition, for PM_2.5_, PM_10_ and CO, stronger associations were typically observed in the high-pollution (2014) and low-pollution (2018/2019) periods.

Skin diseases have become a severe public health concern owing to the high prevalence and steadily increasing disease burden [7]. Apart from the respiratory system, the skin is one of the major target organs of ambient air pollutants [16,32]. PM is the carrier of multiple noxious constituents, including heavy metal elements and organic components, which is in direct contact with skin and might be enriched in its surface [32]. Gaseous air pollutants are not considered to exert direct effects on skin, but might readily interact with molecules in the stratum corneum [33]. The potential underlying mechanisms mediating the effects of air pollutants on skin diseases remain unclear, but metabolism, inflammatory processes and oxidative stress may play important roles [16]. For instance, short-term exposure to air pollutants within days or even hours might directly and indirectly induce inflammatory status, reactive oxygen species (ROS) level, lipid peroxidation and protein oxidation in skin, resulting in the onset or exacerbation of skin diseases [16,34]. In this study, short-term exposure to some pollutants (e.g., PM_2.5_ and NO_2_) were positively associated with emergency room visits for total skin diseases, dermatitis/eczema and urticaria. Our results were generally similar to previous findings showing that ambient air pollution exposure was positively associated with outpatient visits for dermatitis and eczema [10,11,35], as well as hospital admissions for skin diseases [22].

The subgroup analyses found stronger associations of SO_2_ exposure with urticaria in middle-aged people (≥40 years) compared to younger people (≤18 years). A previous study found that elderly people might be more susceptible to air pollutant–dermatitis/eczema associations [10]. However, this study did not provide stratified analyses solely for the elderly (≥65 years) due to the limited number of cases. Meanwhile, we found stronger associations of urticaria visits with PM_2.5_ and PM_10_ exposure in the cold season. This might be attributable to the PM source. In cold weather, coal combustion and traffic emissions are the major sources of PM, which might induce stronger inflammatory responses [36]. Meanwhile, it is plausible that the cold weather may potentiate the inflammation and oxidative stress when exposed to PM and result in the exacerbation of urticaria [37]. Previous studies found stronger associations between PM and outpatient visits for dermatitis and eczema in the warm season [10,11], but this study did not observe differences between the two groups. As for sex differences, we found that female individuals were more predisposed to PM_2.5_ exposure for urticaria visits. However, a study in Chengdu, China found stronger associations between PM exposure and hospital admission for total skin diseases in male individuals [22]. The inconsistent results above might partly be attributed to the geography and population variations across studies [38]. Meanwhile, aforementioned studies used different clinical outcomes, which could be another reason for the inconsistency. This study used emergency room visits as the outcome measure, which is more sensitive for the characterization of the acute onset of diseases due to urgent need of medical services [39].

The improvement of air quality contributes to reductions in disease burden attributable to air pollutant exposure [20], however, whether it can reduce the risks of the onset and development of diseases is still a controversial issue. A study in Rome, Italy found that the effects of air pollution on mortality were generally unchanged over the last two decades [28]. Similar findings were also reported by studies in Switzerland [40] and Canada [41]. However, decreasing exposure–health associations were found with a background of air quality improvement in Germany [42], the U.S. [43] and Korea [44], and an opposite trend was found in Canada [29].

Since the implementation of APPCAP in 2013, the government of China have initiated a series of air pollution control measures. Consequently, key regions like Beijing showed remarkable decreases of 10~50% in major air pollutants except for O_3_, as shown in our results and previous studies [17,18]. However, no previous studies have examined its effects on the association between air pollution exposure and skin diseases over recent years in China. In this study, we did not observe consistent temporal variability of this association over the whole study period (2014–2019). Interestingly, we found stronger annual associations of some pollutants (e.g., PM_2.5_ and PM_10_) with emergency room visits for total skin diseases and urticaria in the high-pollution (2014) and low-pollution (2018/2019) periods, although the pollutant concentrations were continuously declining over this period. We speculated that this might be partly attributed to changes in pollution sources and components [36]. Due to the implementation of air pollution prevention and control strategies in Beijing, the emissions from fossil fuel combustion (e.g., coal combustion) are considerably decreased, while vehicle emissions account for greater proportion over time [18,45]. Accordingly, the components change with the restructuring of pollution sources [46,47], and thus the exposure–health association might not decrease along with the declining concentrations of air pollutants [28,48,49]. In addition, the changes of demographic distribution and population susceptibility might also be reasons for the findings [28,41].

A strength of the present study is that we provide evidence of the air pollutant–skin disease associations with the background of constantly improving air quality due to the implementation of a series of air pollution prevention and control measures in China. To date, relevant studies are still limited, especially studies in developing countries over a relatively long period. Second, we used emergency room visit data as the outcome measure, which might more sensitively capture the acute effects of air pollutants.

Several limitations of this study should be noted. First, this study is a time-series ecological study, and thus the results should be interpreted cautiously, for association does not imply causation. Meanwhile, using environmental monitoring data as a proxy for individual exposure might include exposure measurement errors. In addition, skin diseases might be attributable to other confounding factors such as health status, allergen exposure or diet and nutrition [50]. However, these factors were not evaluated due to the limitation of the time-series design and data availability. Second, the study cases were derived from one general hospital in Beijing, thus the findings still need further verification at a larger population scale. Third, the skin diseases covered a wide range of specific diseases. The positive associations of air pollutants with total skin diseases do not necessarily mean that a variety of skin diseases might be attributable to air pollution exposure. Fourth, this study did not examine the association between PM components and skin diseases owing to the lack of data on PM components, and further studies are needed to test our hypothesis. Lastly, we used multiple testing to evaluate the associations of air pollution with skin diseases, which might lead to potential false positive results.

## 5. Conclusions

We found that short-term exposures to certain air pollutants (e.g., PM_2.5_ and NO_2_) were associated with increased emergency room visits for skin diseases such as dermatitis/eczema and urticaria. Meanwhile, some of the associations were found both in the high-pollution (2014) and low-pollution (2018/2019) periods. Our study emphasizes the necessity of controlling the potential health hazard of air pollutants on skin, although significant achievements in air quality control have been made in China. Further evidence on the associations of sources and components of air pollution with skin diseases is still needed, in order to establish guided environmental health policies at the population level.

## Figures and Tables

**Table 1:** Demographic characteristics of patients included in this study, 2014–2019.

Variable	Total (*N* = 45,094)		Dermatitis/Eczema (*N* = 16,891)		Urticaria (*N* = 23,832)	
	N	%	N	%	N	%
Sex ^a^						
Male	18,251	40.5	6394	37.9	9649	40.5
Female	26,843	59.5	10,497	62.1	14,183	59.5
Age (years) ^a^						
0–18	9022	20.0	3618	21.4	4929	20.7
19–39	25,004	55.4	8958	53.0	13,882	58.2
≥40	11,068	24.5	4315	25.5	5021	21.1
Season						
Cold (November–April)	17,615	39.1	6227	36.9	9761	41.0
Warm (May–October)	27,479	60.9	10,664	63.1	14,071	59.0

^a^ Subgroups excluded cases without sex or age information.

## Data Availability

The datasets generated and analyzed during the current study are not publicly available due to protection of patient privacy, but are available from the corresponding author on reasonable request.

## Supplementary Materials

The following are available online at https://www.mdpi.com/article/10.3390/toxics9050108/s1, Table S1: Summary statistics of daily ambient air pollutants and meteorological variables in Beijing during the study period (2014–2019). Table S2: Spearman correlation coefficients among the ambient air pollutants and meteorological variables in Beijing during the study period (2014–2019). Table S3: Associations between ambient air pollutants and daily emergency room visits for skin diseases in two-pollutant models. Table S4: Associations between ambient air pollutants and daily emergency room visits for skin diseases in sensitive analyses in single-pollutant models. Figure S1: Daily emergency room visits for skin diseases during the study period (2014–2019). Figure S2: Percent changes and 95% confidence intervals (CIs) in daily emergency room visits for skin diseases per 10 μg/m^3^ increases in PM_2.5_, PM_10_, 8 h maximum O_3_, SO_2_, NO_2_ and 1 mg/m^3^ increase in CO with different time metrics from 2014–2019, stratified by age. Figure S3: Percent changes and 95% confidence intervals (CIs) in daily emergency room visits for skin diseases per 10 μg/m^3^ increases in PM_2.5_, PM_10_, 8 h maximum O_3_, SO_2_, NO_2_ and 1 mg/m^3^ increase in CO with different time metrics from 2014–2019, stratified by season. Figure S4: Percent changes and 95% confidence intervals (CIs) in daily emergency room visits for skin diseases per 10 μg/m^3^ increases in PM_2.5_, PM_10_, 8 h maximum O_3_, SO_2_, NO_2_ and 1 mg/m^3^ increase in CO with different time metrics from 2014–2019, stratified by sex. Figure S5: Percent changes and 95% confidence intervals (CIs) in daily emergency room visits for skin diseases along with a 10 μg/m^3^ increases in PM_2.5_ (lag 0–5), PM_10_ (lag 0–3), 8 h maximum O_3_ (lag 0–5), SO_2_ (lag 0–6), NO_2_ (lag 0–1) and a 1 mg/m^3^ increase in CO (lag 0–5) from 2014–2015, 2016–2017 and 2018–2019. Figure S6: The exposure–response curve for the association between ambient air pollutant concentrations and risk for emergency room visits for skin diseases during the study period (2014–2019).
