# Supplementary material for: Short-Term Exposure to Ambient Air Pollution and Increased Emergency Room Visits for Skin Diseases in Beijing, China
Source: Toxics. 2021 May 12;9(5):108. doi: 10.3390/toxics9050108 (PMC8151157; doi:10.3390/toxics9050108)
Supplement: Supplementary file 1 [file toxics-09-00108-s001.zip › toxics-1188951-supplementary.pdf]

Supplementary Materials: Short-Term Exposure to Ambient Air Pollution and Increased Emergency Room Visits for Skin Diseases in Beijing, China

Wanzhou Wang, Wenlou Zhang, Jingjing Zhao, Hongyu Li, Jun Wu, Furong Deng, Qingbian Ma and Xinbiao Guo

Table S1. Summary statistics of daily ambient air pollutants and meteorological variables in Beijing during the study period (2014–2019).

Variable	Mean	SD	Minimum	5th	Median	95th	Maximum	IQR (25th–75th)
$PM_{2.5}$ (μg/m^3)	64.2	59.4	3.0	9.0	47.0	185.8	477.0	60.0 (24.0–84.0)
PM_{10} (μg/m^3)	91.5	66.7	7.0	23.0	75.0	221.0	550.0	73.0 (45.0–118.0)
8 h-maximum O_3 (μg/m^3)	98.1	62.8	2.0	14.0	83.0	220.0	311.0	88.0 (52.0–140.0)
SO_2 (μg/m^3)	10.2	13.6	2.0	2.0	5.0	37.4	133.0	8.0 (3.0–11.0)
NO_2 (μg/m^3)	45.5	22.5	6.0	18.0	40.0	92.0	155.0	26.8 (30.0–56.8)
CO (mg/m^3)	1.0	0.8	0.2	0.3	0.8	2.7	8.0	0.6 (0.6–1.2)
Temperature (°C)	13.9	11.2	−14.3	−3.1	15.3	29.0	32.6	21.5 (2.8–24.3)
RH (%)	50.8	19.8	8.0	19.4	51.0	83.3	99.0	31.5 (35.0–66.5)

Abbreviations: SD = Standard deviation; IQR = Interquartile range; RH = Relative humidity.

Table S2. Spearman correlation coefficients among the ambient air pollutants and meteorological variables in Beijing during the study period (2014–2019).

Variable	$PM_{2.5}$	PM_{10}	8 h-maximum O_3	SO_2	NO_2	CO	Temperature	RH
$PM_{2.5}$	—	0.86 **	−0.03	0.56 **	0.71 **	0.87 **	−0.04	0.49 **
PM_{10}		—	0.01	0.60 **	0.71 **	0.70 **	−0.05	0.23 **
8 h maximum O_3			—	−0.28 **	−0.35 **	−0.24 **	0.81 **	0.04
SO_2				—	0.66 **	0.63 **	−0.47 **	−0.16 **
NO_2					—	0.75 **	−0.30 **	0.22 **
CO						—	−0.20 **	0.49 **
Temperature							—	0.31 **
RH								—

Abbreviations: RH = Relative humidity. * $p < 0.05$, ** $p < 0.01$.

Table S3. Associations between ambient air pollutants and daily emergency room visits for skin diseases in two-pollutant models.

Indicator	Two-Pollutant Model with $PM_{2.5}$	Two-Pollutant Model with PM_{10}	Two-Pollutant Model with 8 h-maximum O_3	Two-Pollutant Model with SO_2	Two-Pollutant Model with NO_2	Two-Pollutant Model with CO
**Total skin disease**						
$PM_{2.5}$ (lag 0–5)	—	**2.1 (0.8, 3.4)** *	**0.9 (0.2, 1.5)** *	**0.8 (0.1, 1.5)** *	0.8 (−0.1, 1.7)	**1.2 (0.4, 2.1)** *
NO_2 (lag 0–1)	0.3 (−1.3, 1.9)	1.4 (−0.2, 3.0)	1.1 (0.0, 2.3) *	0.7 (−0.7, 2.1)	—	**2.3 (0.7, 4.0)** *
**Dermatitis/Eczema**						
$PM_{2.5}$ (lag 0–1)	—	**2.2 (0.9, 3.5)** *	**1.2 (0.5, 1.9)** *	**1.0 (0.2, 1.7)** *	0.9 (0.0, 1.8)	**1.7 (0.8, 2.6)** *
PM_{10} (lag 0–3)	−0.7 (−2.0, 0.6)	—	0.5 (−0.2, 1.2)	0.7 (0.0, 1.5)	0.5 (−0.4, 1.5)	**1.1 (0.2, 1.9)** *
O_3 (lag 0–5)	1.1 (−0.2, 2.3)	**1.4 (0.2, 2.6)** *	—	**1.4 (0.3, 2.5)** *	**1.3 (0.2, 2.5)** *	**1.4 (0.3, 2.5)** *
NO_2 (lag 0–1)	0.8 (−1.7, 3.4)	**2.6 (0.0, 5.2)** *	**2.6 (0.7, 4.5)** *	2.1 (−0.1, 4.4)	—	**3.8 (1.2, 6.5)** *

Indicator	Two-Pollutant Model with $PM_{2.5}$	Two-Pollutant Model with PM_{10}	Two-Pollutant Model with 8 h-maximum O_3	Two-Pollutant Model with SO_2	Two-Pollutant Model with NO_2	Two-Pollutant Model with CO
**Urticaria**						
O_3 (lag 0–2)	−0.6 (−1.3, 0.1)	−0.5 (−1.2, 0.2)	—	−0.5 (−1.1, 0.2)	−0.5 (−1.1, 0.2)	−0.4 (−1.1, 0.2)
SO_2 (lag 0–6)	**6.6 (0.3, 13.3)** *	**7.9 (1.6, 14.5)** *	**8.6 (3.2, 14.3)** *	—	**6.6 (0.1, 13.6)** *	**8.1 (1.8, 14.8)** *
NO_2 (lag 0–5)	1.4 (−1.6, 4.6)	2.7 (−0.4, 5.8)	**2.7 (0.6, 4.9)** *	1.2 (−1.4, 3.9)	—	**3.4 (0.2, 6.7)** *

Note: Two-pollutant models were conducted only for the most significant effect estimate of each air pollutant (with the smallest *p*-value) by controlling for another air pollutant with the same time metrics. Data are shown as percent changes and 95% confidence intervals (CIs). * $p < 0.05$.

Table S4. Associations between ambient air pollutants and daily emergency room visits for skin diseases in sensitive analyses in single-pollutant models.

Indicator	Sensitive Estimates [a]
**Total Skin Disease**	
$PM_{2.5}$ (lag0–5)	**0.8 (0.2, 1.4) ***
NO_2 (lag0–1)	0.8 (−0.4, 1.9)
**Dermatitis/Eczema**	
$PM_{2.5}$ (lag0–1)	**1.0 (0.3, 1.6) ***
PM_{10} (lag0–3)	**0.7 (0.0, 1.3) ***
O_3 (lag0–5)	**1.5 (0.4, 2.5) ***
NO_2 (lag0–1)	**2.3 (0.5, 4.3) ***
**Urticaria**	
O_3 (lag0–2)	−0.5 (−1.1, 0.2)
SO_2 (lag0–6)	**8.6 (3.0, 14.4) ***
NO_2 (lag0–5)	**2.4 (0.3, 4.6) ***

[a] Single-pollutant models adjusted for day of week, holiday, calendar time, distributed lag nonlinear model (DLNM) with a maximum period of 30 days and 4 degrees of freedom for temperature, and humidity. * $p < 0.05$.

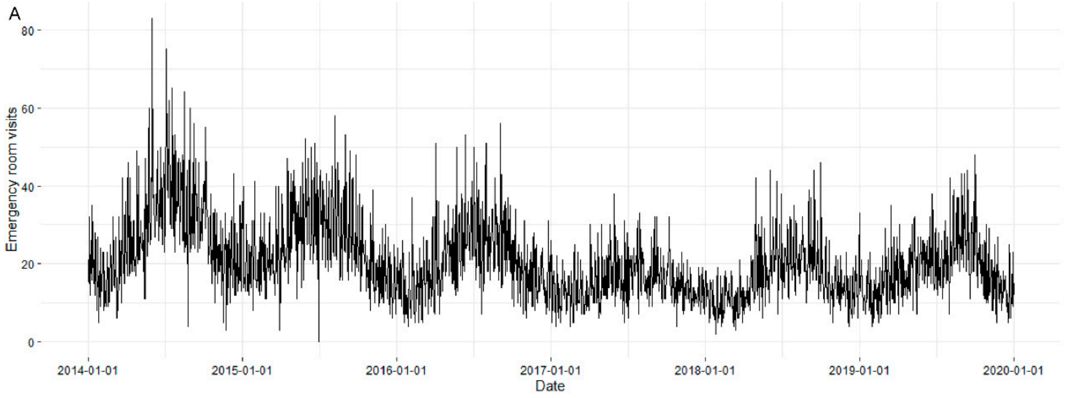

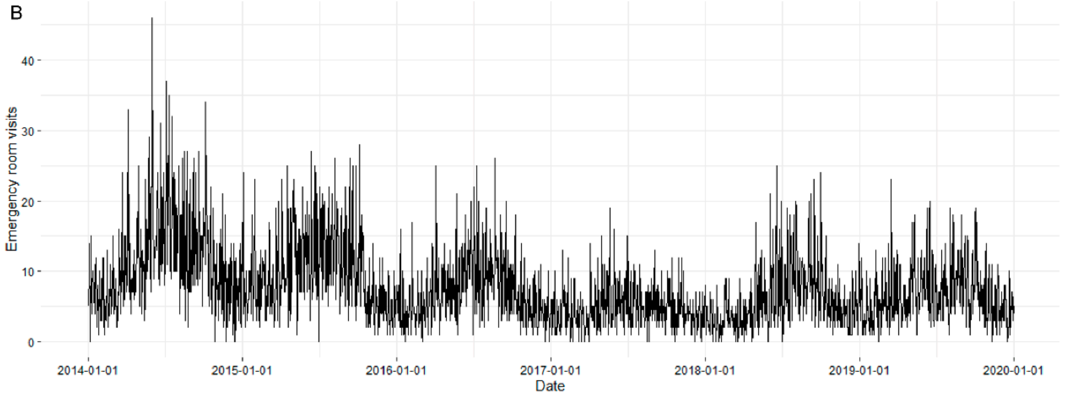

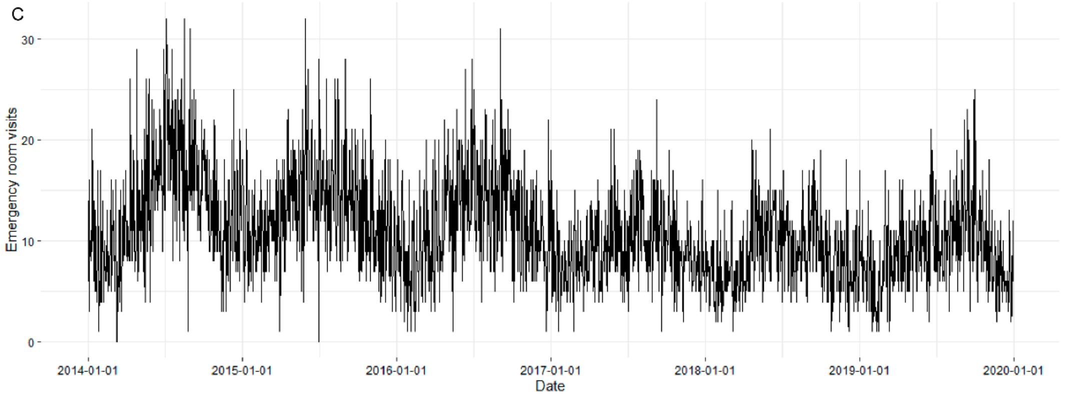

Figure S1. Daily emergency room visits for skin diseases during the study period (2014–2019). (**A**) Total emergency room visits for skin diseases; (**B**) emergency room visits for dermatitis/eczema; (**C**) emergency room visits for urticaria.

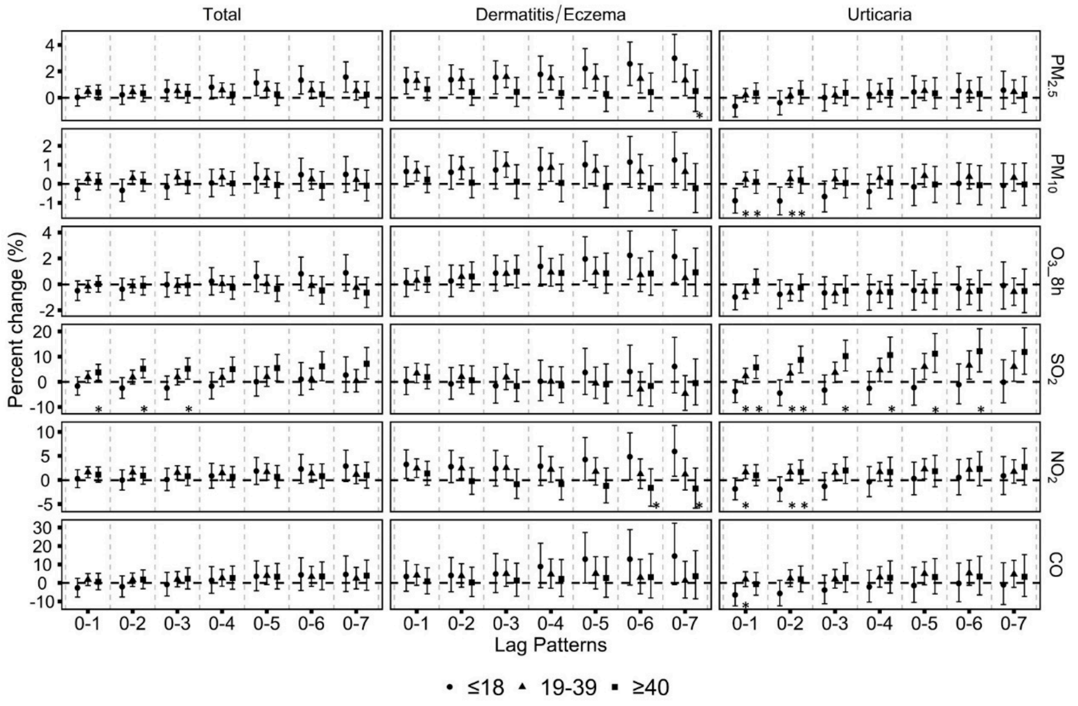

Figure S2. Percent changes and 95% confidence intervals (CIs) in daily emergency room visits for skin diseases per 10 μg/m³ increases in $PM_{2.5}$, PM_{10}, 8 h maximum O_3, SO_2, NO_2 and 1 mg/m³ increase in CO with different time metrics from 2014–2019, stratified by age.

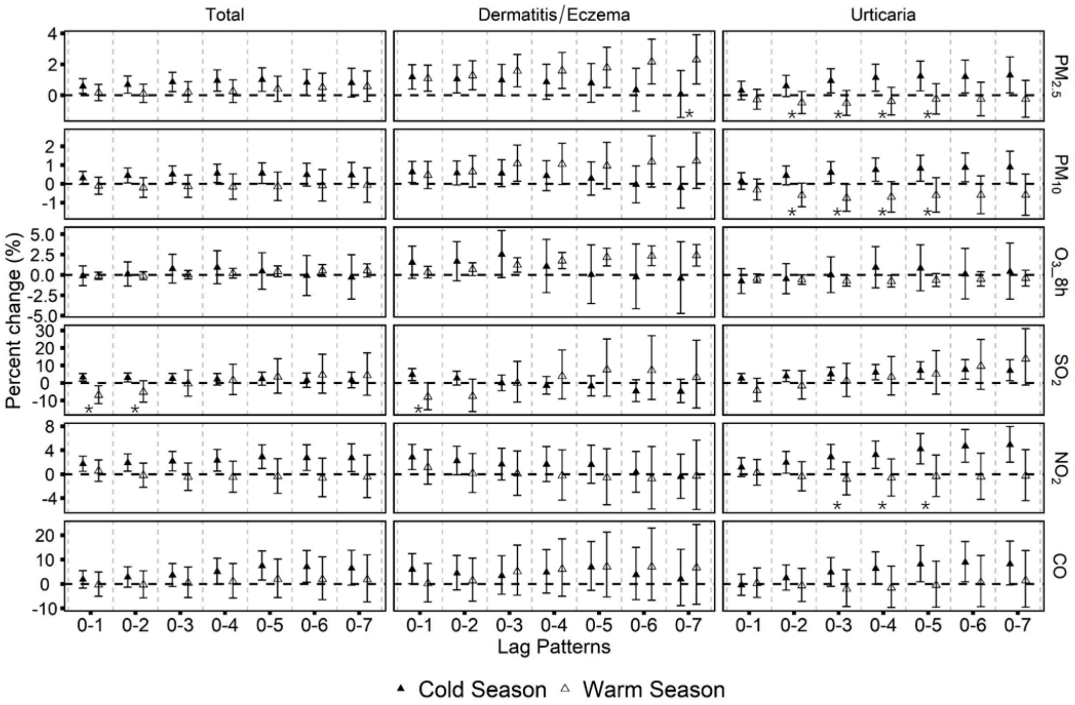

Figure S3. Percent changes and 95% confidence intervals (CIs) in daily emergency room visits for skin diseases per 10 μg/m^3 increases in $PM_{2.5}$, PM_{10}, 8 h maximum O_3, SO_2, NO_2 and 1 mg/m^3 increase in CO with different time metrics from 2014–2019, stratified by season. Note: Warm season: May–October; cold season: Nov-Apr. * p for subgroup differences < 0.05.

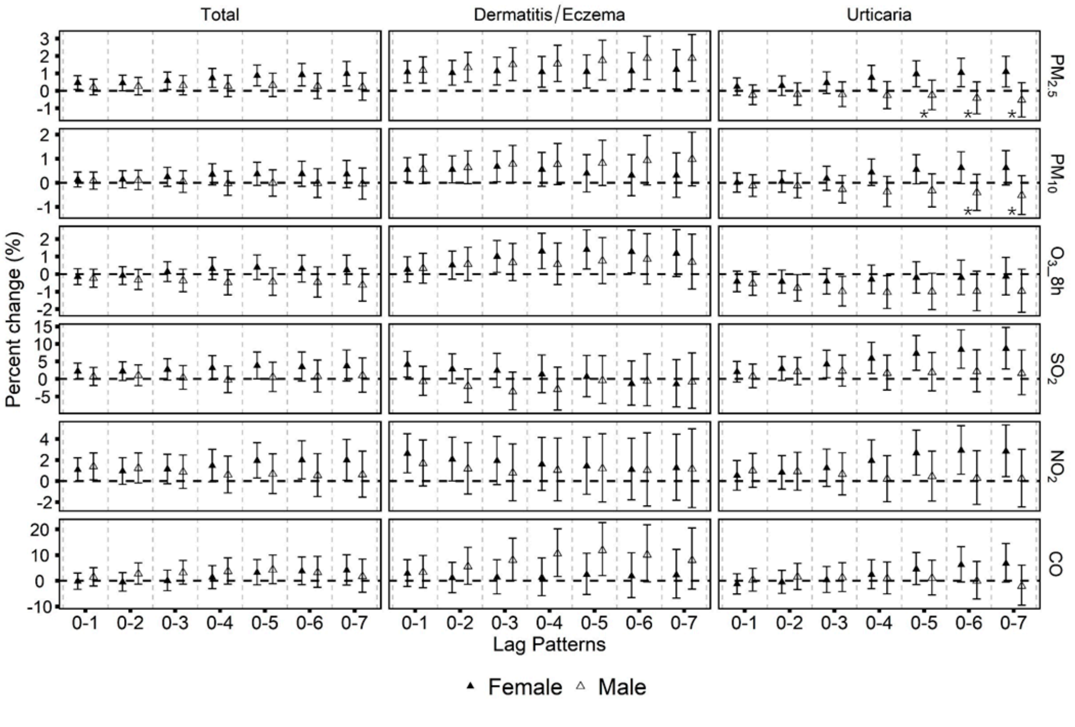

Figure S4. Percent changes and 95% confidence intervals (CIs) in daily emergency room visits for skin diseases per 10 μg/m³ increases in $PM_{2.5}$, PM_{10}, 8 h maximum O_3, SO_2, NO_2 and 1 mg/m³ increase in CO with different time metrics from 2014–2019, stratified by sex. * *p* for subgroup differences < 0.05.

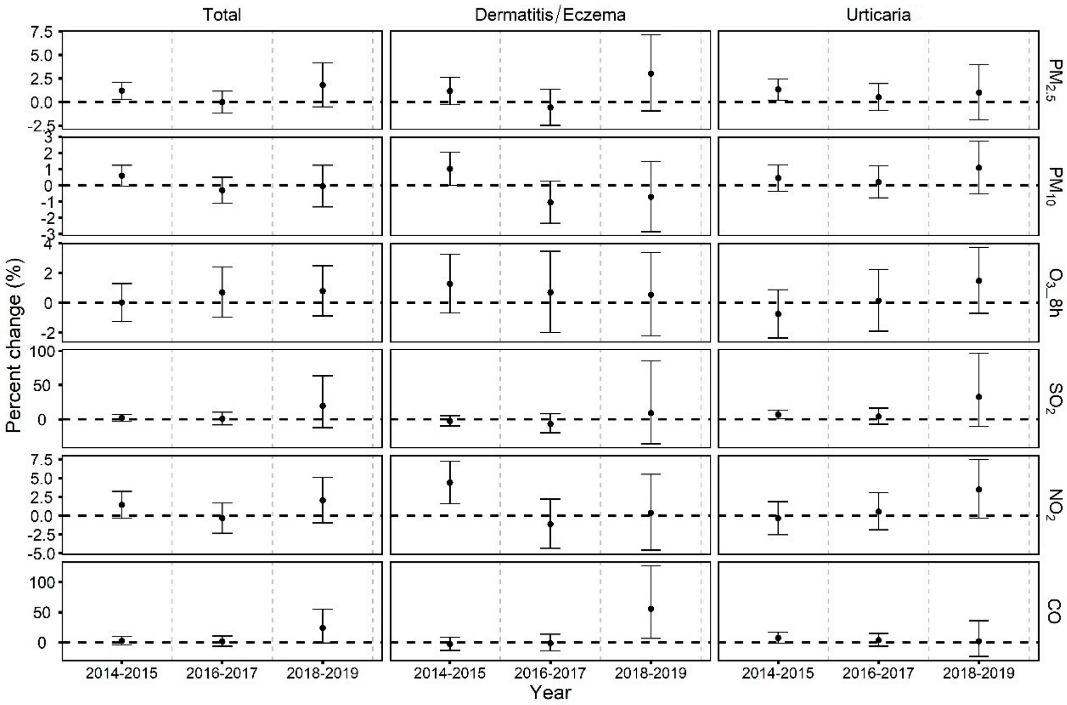

Figure S5. Percent changes and 95% confidence intervals (CIs) in daily emergency room visits for skin diseases along with a 10 μg/m³ increase in $PM_{2.5}$ (lag 0–5), PM_{10} (lag 0–3), 8 h maximum O_3 (lag 0–5), SO_2 (lag 0–6), NO_2 (lag 0–1) and a 1 mg/m³ increase in CO (lag0–5) from 2014–2015, 2016–2017 and 2018–2019.

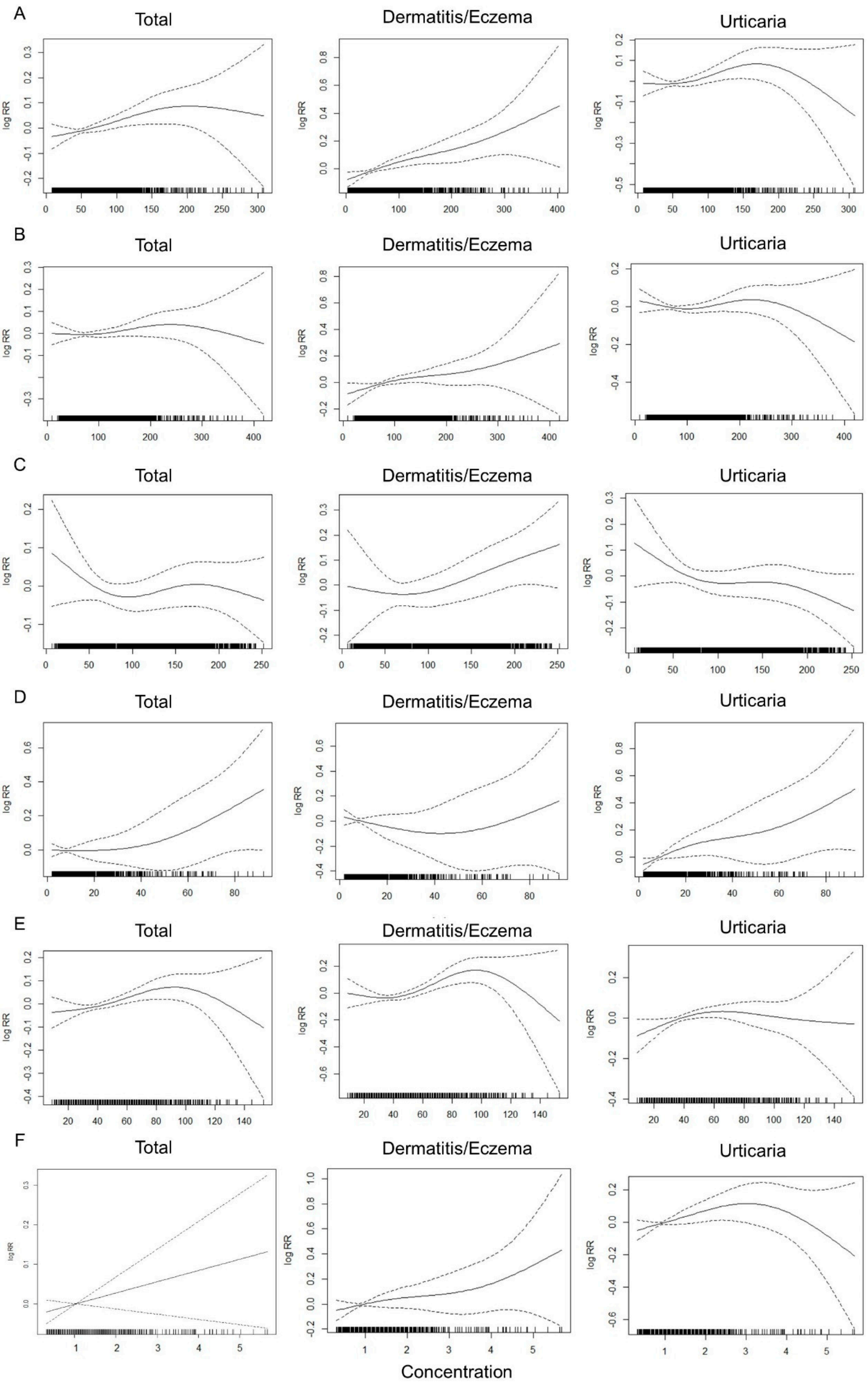
A
Total
Dermatitis/Eczema
Urticaria
B
Total
Dermatitis/Eczema
Urticaria
C
Total
Dermatitis/Eczema
Urticaria
D
Total
Dermatitis/Eczema
Urticaria
E
Total
Dermatitis/Eczema
Urticaria
F
Total
Dermatitis/Eczema
Urticaria
log RR
Concentration

Figure S6. The exposure–response curve for the association between ambient air pollutant concentrations and risk for emergency room visits for skin diseases during the study period (2014–2019). (**A**) $PM_{2.5}$ (lag 0–5), (**B**) PM_{10} (lag 0–3), (**C**) 8 h maximum O_3 (lag 0–5), (**D**) SO_2 (lag 0–6), (**E**) NO_2 (lag 0–1) and (**F**) CO (lag 0–5).
